# PB.14. An analysis of subtle alterations in reading pattern with the introduction of digital mammography screening: South East Scotland Breast Screening Programme

**DOI:** 10.1186/bcr3706

**Published:** 2014-11-03

**Authors:** G Babu, A Gilchrist, J Murray

**Affiliations:** 1 NHS Lothian, Edinburgh, UK

## Introduction

Digital screening was implemented in this programme in November 2013. Following an impression that the pattern of recalls had changed, this study aimed to identify cases recalled for subtle or vague mammographic features which were true lesions and those where there was overcall, and perhaps suggest guidance for readers.

## Methods

A total of 235 recall cases were randomly identified between November 2013 and March 2014. All were possible subtle lesions, parenchymal deformity or asymmetry. Definite opacities, calcifications and symptoms were excluded. The cases were reviewed by two experienced film readers and a specialist trainee and categorised as Category 1, definite overcall; Category 2, Possible overcall; category 3, definite recall.

## Results **and conclusion**

Following assessment, all cases in Category 1 were composite normal breast tissue (NBT) and returned to normal routine recall. In Category 2, 37 were composite NBT; 19 were real lesions, mostly cysts; four were cancers. In Category 3, 51 were real lesions, mostly cysts; 17 were cancers. This study has demonstrated that in Category 1 cases (definite overcall), no cancers would have been missed. In Category 2 cases (possible overcall), we must accept some overcall as this group can hide subtle cancers. In Category 3 cases (definite recall), almost all warranted recall. The cancers appeared suspicious on screening mammograms. The difference in feature perception between digital and analogue mammography is significant and may alter recall practice. Critical analysis of subtle appearances is required to prevent overcall for future readers.

